# Retinal bipolar cells borrow excitability from electrically coupled inhibitory interneurons to amplify excitatory synaptic transmission

**DOI:** 10.1101/2024.07.03.601922

**Published:** 2024-07-05

**Authors:** Shubhash Chandra Yadav, Logan Ganzen, Scott Nawy, Richard H Kramer

**Affiliations:** 1. University of California Berkeley, Department of Molecular and Cell Biology. Berkeley, CA, USA

## Abstract

Bipolar cells of the retina carry visual information from photoreceptors in the outer retina to retinal ganglion cells (RGCs) in the inner retina. Bipolar cells express L-type voltage-gated Ca^2+^ channels at the synaptic terminal, but generally lack other types of channels capable of regenerative activity. As a result, the flow of information from outer to inner retina along bipolar cell processes is generally passive in nature, with no opportunity for signal boost or amplification along the way. Here we report the surprising discovery that blocking voltage-gated Na^+^ channels profoundly reduces the synaptic output of one class of bipolar cell, the type 6 ON bipolar cell (CBC6), despite the fact that the CBC6 itself does not express voltage-gated Na^+^ channels. Instead, CBC6 borrows voltage-gated Na^+^ channels from its neighbor, the inhibitory AII amacrine cell, with whom it is connected via an electrical synapse. Thus, an inhibitory neuron aids in amplification of an excitatory signal as it moves through the retina, ensuring that small changes in the membrane potential of bipolar cells are reliably passed onto downstream RGCs.

## INTRODUCTION

In most brain neurons, action potentials invade synaptic terminals to generate large depolarizations that ensure robust release of neurotransmitter. In contrast, many retinal neurons employ graded potentials to control synaptic output, enabling release to be finely tuned. However, there is a trade-off. Without a mechanism for amplifying small voltage signals, synaptic output can be obscured, limiting detection by the postsynaptic cell and the remainder of the visual system.

The rod pathway solves this problem in several ways. For example, in the rod pathways, convergence of many rods onto fewer rod bipolar cells (RBCs) and many RBCs onto still fewer AII amacrine cells, aids in signal amplification (Deans et al., 2002; Volgyi et al., 2004; Dunn et al., 2006). At the RBC input synapse, the rate of glutamate release from rods in darkness is sufficient to saturate mGluR6 receptors such that small fluctuations in glutamate release are insufficient to alter mGluR6 signaling and trigger a postsynaptic response. At converging input synapses onto an RBC, this nonlinear thresholding mechanism filters out noise while allowing reliable detection of small light-elicited synaptic events that occur simultaneously in multiple rods (Field and Rieke, 2002; Sampath and Rieke, 2004). Most BCs synapse directly onto RGCs, but the at the RBC output synapse the signals are rerouted through AII amacrine cells, which synapse on cone bipolar cell terminals, employing gap junctions to electrically transmit their signal with minimal delay. AII amacrine cells have voltage-gated Na^+^ channels which accelerate and boosts the effect of voltage changes driven by synaptic input from RBCs. However, it is unclear whether voltage gated sodium channels play any role in transmission at the AII output synapse (Tian et al., 2010).

Here we address a novel mechanism employed by the cone pathway to solve this problem. The cone pathway exhibits much less convergence than the rod pathway, making signal amplification at each synapse more important. Many types of BCs in fish retina express voltage-gated sodium channels (Zenisek et al., 2001), but only rarely in BCs of mammalian retina (Puthussery et al., 2013). Unlike the rod pathway, which reroutes its signal through excitable AII amacrine cells, the cone pathway involves direct synaptic transmission between cone bipolar cells and RGCs. Most of the cone ON-BCs also establish an electrical synapse with AII amacrine cells, but the function of this connection has been unclear.

Analysis of the rod and the cone pathways has been limited by the interconnectedness of retinal circuitry, which makes it difficult to study individual synaptic pathways in isolation. We have employed an optogenetic strategy to selectively stimulate one type of ON-bipolar cell which has an electrical connection with AII cells and also has direct synaptic output onto On-alpha RGCs, the most prevalent RGC type in the mouse retina. Our experiments show that membrane excitability of AII cells, provided by their voltage-gated sodium channels, is “borrowed” by the CBC6 cells to amplify their synaptic output, dramatically potentiating excitatory synaptic transmission to RGCs.

## RESULTS

### Role of voltage-gated Na^+^ channels in amplifying synaptic output of On-BCs

**Figure 1.**
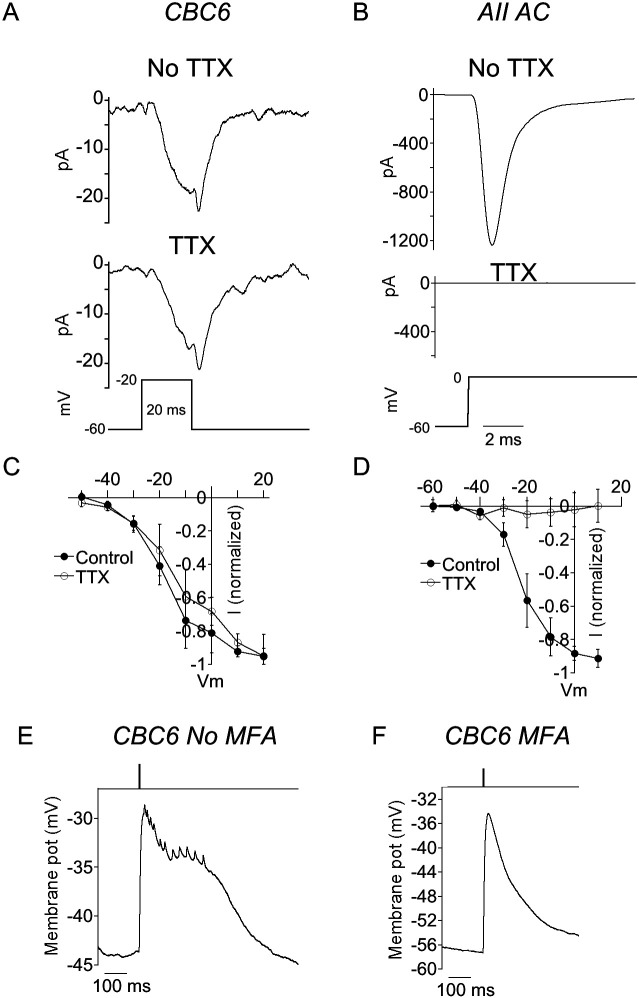
AII amacrine, but not CBC6 cells express TTX-sensitive voltage-gated currents. A, B) Recording of an inward current elicited by a depolarizing voltage step (bottom trace) in a CBC6 (A) and an AII amacrine cell (B) before (top) and during (middle) TTX application. C, D) Summary current voltage plots in the absence and presence of TTX in CBC6 (C) and AII cells (D). n=4 CBC6 and 5 AII cells. E) Recording from a CBC6 during optogenetic stimulation of the recorded and nearby CBC6s. F) Same cell as in (E) but in the presence of MFA.

To search for a potential role of voltage-gated Na^+^ channels in augmenting transmitter release from BCs, we made retinal slices and recorded voltage-gated currents under whole cell patch clamp. We targeted type 6 cone BCs (CBC6), because there are cell-specific promoters that enable selective expression of optogenetic tools in these cells, and because it’s postsynaptic partner, the ON α-RGC (Schwartz et al., 2012), is readily identified based on soma size and dendritic stratification. We used a mouse strain in which CBC6 expresses a ChR2-EYFP fusion protein, allowing for both identification and optogenetic stimulation. Depolarizing voltage steps from a holding potential of −60 mV elicited inward currents (Fig 1A, top panel), attributable to L-type Ca^2+^ channels, which are widely expressed in BCs and play a major role in evoking transmitter release (Van Hook et al., 2019). Application of the voltage-gated Na^+^ channel blocker tetrodotoxin (TTX) had no effect on this current (Fig 1A, bottom panel), consistent with this view. Currents activated over a range of voltage steps were unchanged by TTX (Fig 1C). We conclude that there is no detectible voltage-gated Na^+^ current in CBC6 cells.

In addition to having an output synapse onto RGCs, CBC6 also connect with AII amacrine cells via gap junctions, localized on the CBC6 synaptic terminal in the ON-lamina of the inner plexiform layer (IPL) (Mills and Massey, 1995; Bloomfield and Dacheux, 2001). Could regenerative activation of voltage-gated Na^+^ channels in electrically connected AII cells contribute to the depolarization of CBC6 terminals? We first confirmed that AIIs are capable of generating Na^+^ spikes (Boos et al., 1993; Wu et al., 2011). AII cells in retinal slices were patch clamped and dye filled to confirm their identity. Depolarizing steps evoked large inward currents that were completely blocked by TTX (Fig 1B, D), confirming that they were mediated by voltage-gated Na^+^ channels. We next returned to current clamp recording of CBC6s. Optogenetic stimulation produced robust depolarizations with spikelets superimposed on the envelope of depolarization (Fig 1E). Such spikelets were not observed in the presence of the gap junction uncoupling agent meclofenamic acid (MFA) (Fig 1F). These results suggest that Na^+^ currents originating in AII cells can be detected at CBC6 soma, the site of the whole cell recording, and might possibly be larger near the axon terminal, the location of the gap junction.

**Figure 2.**
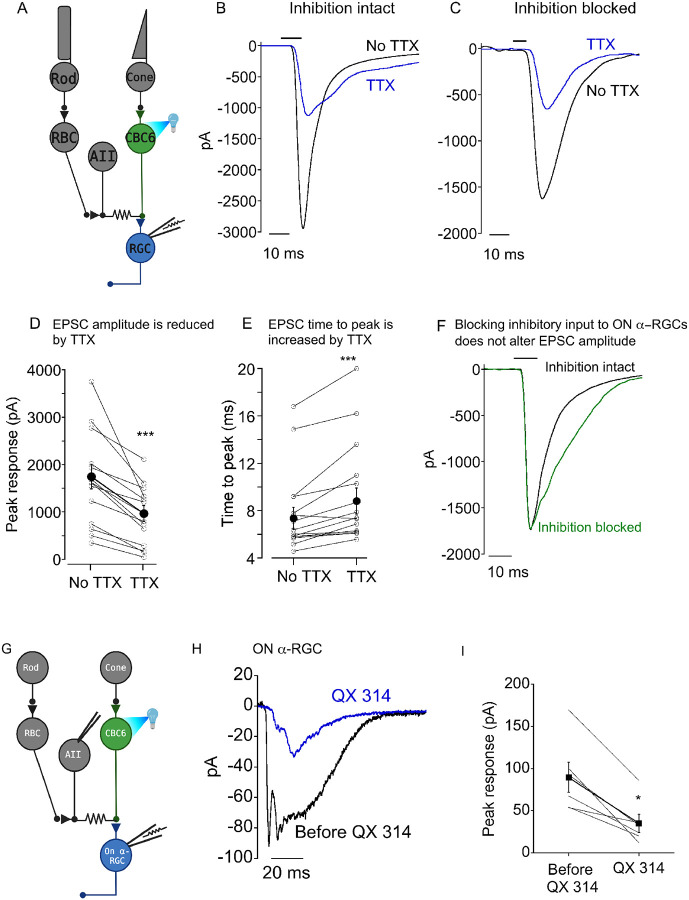
Na^+^ channels amplify synaptic strength and speed kinetics of the CBC output synapse. A) Diagram of experimental setup. EPSCs are driven by optogenetically depolarizing CBC6 bipolar cells while simultaneously recording from downstream ON α-RGCs. B) Representative traces from the same RGC before and during bath application of 500 nM TTX. C) As in (B) except that blockers of GABA, GABA_C_, and glycine receptors were present in the bath. D,E) Summary of the effect of TTX on EPSC amplitude (D) and time to peak (E) in individual RGCs. Filled symbols indicated mean and sem. *** indicates p=0.0001. F) Traces from a single cell showing that blockers of inhibitory synaptic transmission had no effect on EPSC amplitude but slowed the decay. G) Diagram of slice preparation demonstrating simultaneous whole-cell patch clamp of ON α-RGC and intracellular sharp electrode injection of QX 314 in a nearby AII. EPSCs were evoked by optogenetic stimulation of CBC6 as before. H) Example of an ON α-RGC EPSC before and during injection of QX 314 into an AII. I) Summary plot showing the decrease in EPSC amplitude following QX 314 injection. *: p=0.01. N=6.

We wondered if the voltage-gated Na^+^ current in AII amacrine cells might play a role in enhancing synaptic transmission from CBC6 to ON α-RGCs. To test this, we made whole cell recordings from these RGCs in flat mount retina and optogenetically stimulated presynaptic CBC6s (Fig 2A). We found that TTX reduced the amplitude of optogenetically evoked EPSCs by 49±5% and slowed the time to peak by 14±3% (Fig 2B-E).

In addition to AII amacrine cells, which are narrow-field, the retina has many types of wide-field amacrine cells (wf-ACs) that also express voltage-gated Na^+^ channels that are TTX-sensitive (Cook and Werblin, 1994; Bloomfield, 1996). It seemed possible that the effect of TTX on synaptic transmission from CBC6 did not involve voltage-gated Na^+^ channels in AII cells, but instead involved Na^+^ channels in one or more wf-ACs, which make direct chemical inhibitory synapses onto ON α-RGCs. However, blocking chemical inhibition with antagonists of glycine, GABA_A_ and GABA_C_ receptors (strychnine, gabazine and TPMPA) did not alter the peak nor the rise time of the optogenetic EPSC but it did slow the decay of the EPSC (Fig 2F). This is consistent with optogenetic stimulation of CBC6s recruiting wf-ACs through a polysynaptic pathway that exerts feedback inhibition onto bipolar cell terminals with a delay (Eggers and Lukasiewicz, 2006). Furthermore, TTX reduced EPSCs to the same extent whether or not chemical inhibition was blocked (Fig 2B, C). Taken together, these results suggest that wf-ACs made little contribution to rapid synaptic transmission from CBC6 cells, although we cannot rule more delayed effects.

To further implicate AIIs as the source of voltage-gated Na^+^ channels that amplify CBC6 output, we recorded optogenetically evoked EPSCs from ON α-RGCs in retinal slices. A sharp electrode with an internal solution containing the Na^+^ channel blocker QX 314 (5 mM) and the fluorescent dye Alexa 594 was then inserted into a neighboring AII amacrine cell (Fig 2G). The area of the stimulus was restricted to bipolar cells directly over the AII and ON α-RGC. After waiting 5 minutes for diffusion of the internal solution into the AII, EPSCs were once again evoked. In every case, the amplitude of the EPSC was substantially decreased (Fig 2H, I). Responses remained unchanged in amplitude when QX 314 was omitted from the sharp electrode internal solution (data not shown). Thus, regardless of whether the blockade of voltage-dependent Na^+^ channels was global (bath-applied TTX) or specifically targeted to AII cells (intracellular QX 314), the synaptic output of CBC6s was similarly reduced.

### Gap junctional coupling is essential for Na^+^-channel dependent amplification of ON-BCs synaptic output.

**Figure 3.**
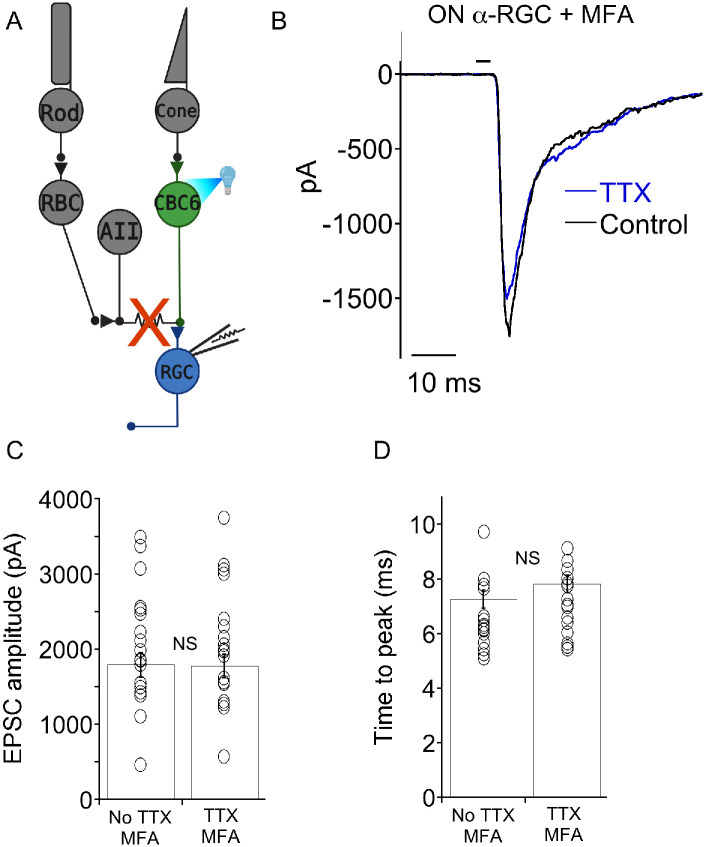
Blocking gap junctions prevents voltage-gated Na^+^ channel-mediated amplification of EPSCs. A) Circuit diagram depicting the cross-over inhibitory feedback in the inner retina. Optogenetically activated CBC6 cells synapse electrically with AII cells via gap junctions. AII cells have chemical synpases onto CBC2 cells, to inhibition of OFF α-RGCs via a glycinergic synapse.B) Recording of IPSCs from an OFF sustained α-RGC that were generated by optogenetic stimulation of CBC6. Application of MFA eliminated IPSCs by blocking spread of current across gap junctions. C) After 30 minute exposure to MFA, application of TTX had no effect on EPSC amplitude in ON α-RGCs. D) Summary data showing that blocking gap junctions completely eliminates the effect of Na^+^ channel block on EPSC amplitude. ** indicates p=0.003

CBC6 cells communicate with AII amacrine through an electrical synapse in the ON-lamina of the IPL, involving gap junctions. If AII amacrine cells contain the voltage-gated Na^+^ channels that amplify the excitatory synapse of CBC6 cells, amplification should be absent if the gap junctions are uncoupled from one another. To test this prediction, we added MFA (100 μM) to the extracellular saline 30-60 minutes before establishing patch clamp recording. Under these conditions, there was no significant effect of TTX on EPSC amplitude or kinetics (Fig 3B-D).

**Figure 4.**
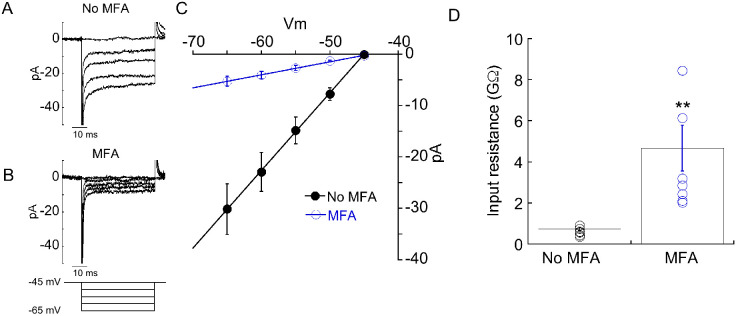
Uncoupling gap junctions with MFA dramatically increases the input resistance of CBC6. A, B) Responses of an CBC6 cell to a series of hyperpolarizing voltage steps before (A) and during (B) application of MFA. C) Average ± sem current-voltage (I-V) relations before (n=7) and during (n=6) MFA application. Fits are from the equation *y=mx+b*, where *m*=slope and *b*=y-intercept. D) Mean and individual CBC6 input resistances, calculated as 1/*m* of each I-V relation, are plotted for each condition. **: p=0.001.

Since MFA uncouples the gap junction which is putatively providing a conduit for voltage-gated Na^+^ current to invade the CBC6 terminal, we hypothesized that peak EPSC amplitudes recorded in the presence of MFA would be reduced due to gap junction uncoupling. However, this was not the case. Overall, the mean peak EPSC amplitude in the absence of MFA was 2.00±0.160 nA (n=32). In the presence of MFA, it was 1.92±0.12 nA (n=30), p=0.85. One potential explanation is that an increase in input resistance resulting from the loss of gap junctional coupling could amplify voltage responses to optogenetic stimulation of CBC6. To test this idea, we measured input resistance of CBC6 cells in the absence and presence of MFA. Starting from a holding potential of −45 mV, we generated hyperpolarizing voltage steps in 5 mV increments (Fig 4A, B). The resulting currents were plotted against voltage and the slope of the I-V plot is the membrane conductance over this span of voltages. In the presence of MFA, the conductance was dramatically reduced (i.e., an increase in membrane resistance) (Fig 4 C, D). We conclude that the electrical junctions between AII and CBC6 cells act to shunt voltage and reduce optogenetic depolarization of CBC6. Voltage gated Na+ channels help compensate for this effect.

**Figure 5.**
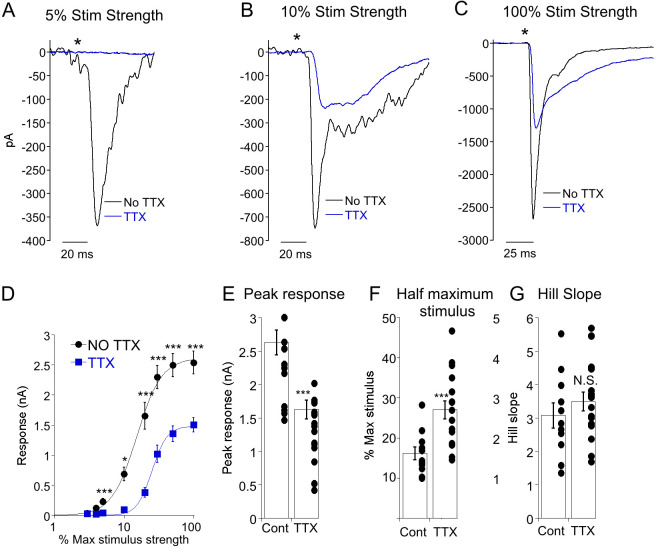
Na^+^ current increases synaptic gain between CBC6 and ON α-RGCs. A-C) ON α-RGC EPSCs elicited by a range of optogenetic stimulus strengths as indicated the presence and absence of TTX. D) Response amplitude plotted as a function of stimulus strength. Maximum stimulus delivered 1000 photons/cm^2^. Continuous lines are the Hill equation fitted to the mean of each group. E-G) Summary of the peak response, half maximal stimulus and Hill slope fitted for each individual cell in the absence and presence of TTX. (E) ***: p=0.0005. (F) ***: p=0.0006.

Because Na^+^ channel activation is voltage-dependent, we wondered if weak optogenetic depolarization of CBC6 would be insufficient to activate Na^+^ channels in electrically coupled AII cells. If weak stimuli were insufficient to activate Na^+^ channels on AIIs, we would anticipate that EPSCs evoked by these stimuli would be insensitive to TTX, and that Na^+^ channel mediated amplification of EPSCs might exhibit a nonlinearity at the stimulus threshold corresponding to Na^+^ channel activation. We therefore evoked EPSCs across a wide range optogenetic stimulus strengths (Fig 5). At 5% of the maximum stimulus strength, EPSC could consistently be evoked in ON α-RGCs under control conditions (Fig 5A). Block of voltage-gated Na^+^ channels typically eliminated EPSCs evoked by weak stimuli, but responses could be readily observed at higher stimulus strengths (Fig 5B, C), a finding that is inconsistent with the idea that voltage-gated Na^+^ channels selectively amplify EPSCs at greater stimulus strengths. Plots of EPSC amplitude as a function of stimulus strength could be well fit by the Hill equation. In the absence of voltage-gated Na^+^ channels, amplitudes displayed both compression and a leftward shift, indicating a loss of synaptic gain (Fig 5D, E, F). Hill slope was unchanged (Fig 5G). These data suggest that Na^+^ channels amplify EPSCs over a wide range of stimulus strengths.

**Figure 6.**
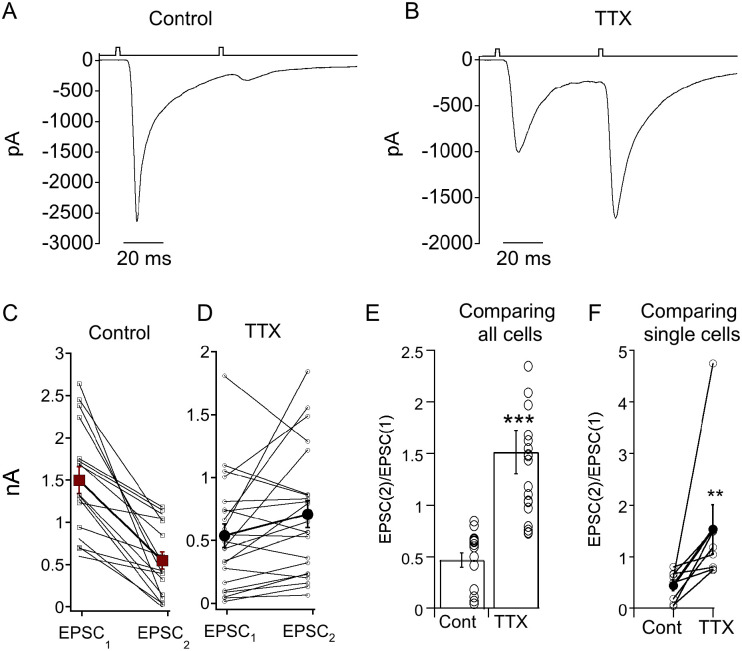
Blocking Na^+^ channels decreases release probably at CBC6 presynaptic terminals. A) Response of an ON α-RGC a pair of stimuli separated by 50 ms. Response to the second stimulus is strongly depressed relative to the first. B) Same cell after bath application of TTX. Responses to the second stimulus is now facilitated relative to the first. C,D) Summary of the response amplitudes to the first (EPSC_1_) and second (EPSC_2_) stimulus in control (C) or TTX (D). Open squares indicate individual cells and filled squares are the means+sem. E) Summary of the paired pulse ratio of all cells measured in control or TTX. *** indicates p<0.0001. F) Paired pulse ratios elicited from a subset of cells in control and TTX. *** indicates p=0.008.

Most synapses exhibit short term presynaptic plasticity. Synapses with high release probability exhibit profound short term depression, while synapses with lower release probability have either more mild short term depression or even short term facilitation (Dittman and Regehr, 1998; Zucker and Regehr, 2002). To determine whether voltage-gated Na^+^ channels boost EPSCs by modulating release probability, we measured paired pulse plasticity at the CBC6/ON α-RGC synapse with and without TTX block. We presented 2 ms flashes with an interval of 50 ms, which is too brief for significant replenishment of released vesicles at BC terminals (von Gersdorff and Matthews, 1997; Singer et al., 2004). Under control conditions, the first flash was sufficient to deplete vesicles sufficiently to result in a strong depression of the second response (Fig 6A). However, in the presence of TTX, the initial response was insufficient to deplete the vesicle pool, and the second stimulus evoked paired pulse facilitation (Fig 6B). This is consistent with the idea that voltage-gated Na^+^ channels potentiate EPSCs by increasing release probability at CBC6 synaptic terminals. The effect of TTX on paired pulse ratio was highly significant when comparing control vs TTX-treated cell populations (Fig 6C), individual cells before and during TTX treatment (Fig 6D).

### Excitability of AII amacrine cells also amplifies crossover inhibition.

**Figure 7.**
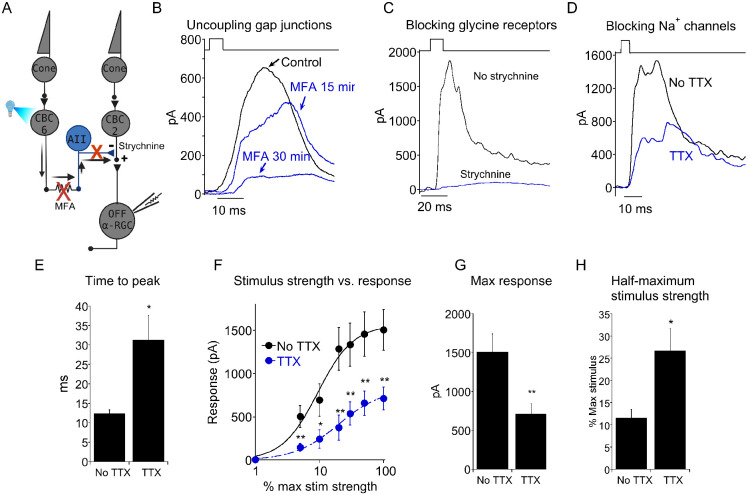
Voltage-gated Na^+^ channels increases the strength of crossover inhibition onto OFF α RGCs. A) Schematic diagram showing signal flow from optogenetic activation of CBC6 to inhibition of OFF α RGCs. B, C) Recording from OFF sustained α-RGC demonstrating that uncoupling of gap junctions with MFA (B) or inhibition of glycine receptors (C) reduces crossover inhibition originating from CBC6 activation. D) Recording from an OFF sustained α-RGC before and during application of TTX. E) Summary histogram demonstrating that blocking voltage-gated Na^+^ channels significantly slowed IPSC time to peak in OFF sustained α-RGCs. N=9 for each group. *: p=0.02. F) Response amplitude plotted as a function of stimulus strength. Maximum stimulus delivered 1000 photons/cm^2^. From weakest to strongest stimulus, **: p=0.008; *: p=0.04; **: p=0.002; **: p=0.004; **: p=0.008; **: p=0.006. Continuous lines are the Hill equation fitted to the mean of each group.

In addition the sign-preserving feed-forward synapse onto ON α-RGCs, CBC6 cells are also engaged in crossover inhibition of OFF α-RGCs. AII amacrine cells are once again crucial for this process. Depolarization of CBC6 spreads to AII amacrine cells through the same gap junctions that pass voltage-gated Na^+^ channel excitation in the opposite direction. When depolarized by optogenetic stimulation of CBC6, AII cells release glycine onto the presynaptic terminals of OFF bipolar cells via a separate set of contacts onto in the OFF-lamina of the IPL (Fig 7A). Under the photopic conditions of our experiments, there is less gap junctional coupling between AII amacrine cells than under scotopic or mesopic conditions (O'Brien and Bloomfield, 2018). We looked for evidence of junctional coupling between AII and BCs by recording from downstream OFF sustained RGCs while optogenetically stimulating CBC6. This produced robust IPSCs in OFF sustained RGCs, providing direct evidence that the gap junctions between AIIs and CBC6s were open sufficiently to allow for conduction. (Fig 7B). These IPSCs were blocked by MFA, consistent with a requirement for gap junctions to propagate this signal to downstream RGCs. Thus we conclude that gap junctional coupling between CBC6 and AIIs is sufficient to support current flow between these cells. As expected, application of strychnine completely blocked the IPSCs (Fig. 7C).

We next tested the possibility that voltage-gated Na+ channels also boost inhibitory synaptic transmission on to OFF sustained RGCs. In support of this, TTX dramatically reduced IPSC amplitude and delayed the time to peak (Fig 7D-G). It also reduced synaptic gain, as demonstrated by a leftward shift in the stimulus strength vs response function (Fig 7F,H). Thus, voltage-gated Na+ channels play two roles, enhancing synaptic output of CBC6 onto the ON pathway, while simultaneously increasing the inhibitory output of AII onto the OFF pathway.

### Dopaminergic modulation of gap junctions also can regulate CBB6 output.

**Figure 8.**
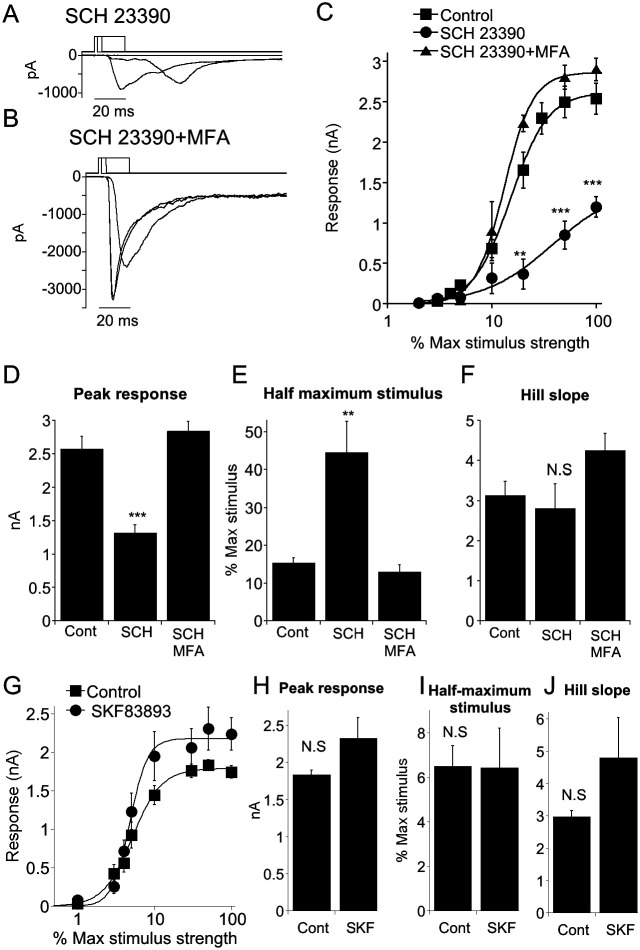
Blocking the D1 receptor depresses synaptic transmission from CBC6s to On α-RGCs, by increasing gap junction coupling between AII and CBC6s. A) Example responses of an ON α-RGC to a series of stimulus strengths, modulated by changing stimulus duration, in the presence of the D1 receptor antagonist SCH 23390. B) As in (A) but with MFA added to the bath. C) EPSC amplitudes plotted as a function of stimulus strength under three conditions. Blockade of gap junctions reversed the strong depressing effect of SCH 23390. Continuous lines are best fits of the Hill equation. Control: n=11. SCH 23390: n=6. SCH 23390+MFA: n=4. *** indicates p=0.0001 vs control. ** indicates p=0.002 vs control. D-F) Summary histograms for Hill parameters to the fits for individual cells. ** indicates p=0.004. G) Summary plot of response vs stimulus strength in the presence and absence of the D1 receptor agonist SKF 83893. Continuous lines are best fits of the Hill equation. H-J) Summary histograms for Hill parameters to the fits for individual cells. SKF 83893 did not significantly alter any of the parameters.

In daylight, dopamine release from closely opposed dopaminergic AC processes reduce gap junction coupling between ACs (Bloomfield and Volgyi, 2009). Increasing coupling between AIIs might result in shunting of the Na^+^ current away from the AII-BC gap junction and reduce synaptic output of CBC6. To test this idea, we bath applied the D1 antagonist SCH 23390 to reduce the effect of endogenously released dopamine on AII-AII junctions. Consistent with this hypothesis, SCH 23390 profoundly depressed synaptic output of CBC6 at all stimulus strengths (Fig 8A, C) compared with control. Addition of MFA in the presence of SCH 23390 restored synaptic output to normal levels (Fig 8B, C). Both the peak response and the stimulus strength required to produce 50% of the peak, obtained from fits of the Hill function, were significantly different from control when D1 receptors were inhibited, but not when gap junctions were blocked by MFA (Fig 8D-F). Addition of TTX in the presence of SCH 23390 had little effect on synaptic output, also consistent with the idea Na^+^ current does not effectively pass through the AII-CB gap junction when AII-AII coupling is enhanced.

## DISCUSSION

AII amacrine cell is arguably the most-studied retinal cell type. However, their electrical synapses with ON cone bipolar cells (ON-CBCs) remains enigmatic. On the one hand, AII uses these gap junction-mediated synapses to feed rod signals to these bipolar cells, and therefore eventually to ON retinal ganglion cells (Deans et al., 2002). This arrangement forms the basis for most-sensitive rod-vision in the majority of vertebrates. On the other hand, the presence of potentially leaky gap junctions on these bipolar cells seems counter-intuitive for processing cone signals.

Therefore, we first investigated whether gap-junction mediated shunting occurs and affects ON-CBCs voltages in the photopic conditions and to what degree. Since a large fraction of ON-CBCs forms gap junctions with AII cells (Tsukamoto and Omi, 2017), a simple trade-off between rod- and cone-pathways is undesirable. This led us to ask another important question: does the retina adopt a strategy to compensate for this effect if present? Our current work largely focuses on these two long-standing questions.

Amongst bipolar cells, type 6 bipolar cells (CBC6s) constitute the majority of gap junctions with AII cells (Tsukamoto and Omi, 2017). Therefore, we used a transgenic mice (cck-cre:ai32) that expresses ChR2 specifically in these cells (Tien et al., 2017) to answer the above questions. We first patched CBC6s in the flat mount light-adapted retina and thus measured their input resistance with and without the application of a gap-junction blocker MFA. Almost five-fold increase in the input resistance of CBC6s after blocking gap junctions (Fig4.) suggests a dramatic shunting-mediated loss of CBC6s conductance when gap-junctions are open. A recent study (Fadjukov et al., 2022) indicated almost 10-fold increase in the input resistance of RPE cells when MFA was applied. However, they showed that both gap junctions and hemichannels contribute to this increase. Therefore, at a current stage, we are unable to completely rule out the possibility that other open channels (pannexins, hemichannels, P2X receptors) may have contributed.

One way to resolve this was to record from the post-synaptic ON-alpha RGCs by optogenetically depolarizing CBC6s and measuring the evoked responses with and without blocking gap junctions. A large increase in synaptic responses from ON-alpha cells would be consistent with the idea that gap-junctions are primarily mediating the observed shunting effect. Indeed, our results confirms this. When CBC6s were depolarized by weak stimulus (<5mV), the EPSC amplitudes measured after the application of 100μM MFA were more than 10 fold larger compared to the control (data not shown). The differences persisted over a wide range of CBC6s’ depolarization. In addition, the stimulus which produced a paired-pulse facilitation in the control consistently produced paired-pulse depression when MFA was superfused. Another gap junction blocker, (β-glycyrrhetinic acid had a similar effect (data not shown). Altogether, these data indicates a dramatic reduction in neurotransmitter release from CBC6s when gap junctions are open. Thus, we conclude that CBC6s and potentially other ON-CBs employing gap junctions heavily shunt their voltages in the photopic conditions.

Yet another convincing support to this conclusion comes from our IPSCs recordings from OFF-sustained alpha cells under the similar conditions. When we depolarized CBC6s, we were able to consistently evoke IPSCs from OFF-sustained alpha cells (Fig 7). These inhibitory responses were almost completely blocked by either gap-junction blocker or glycine receptor antagonist, suggesting that the shunted signals from CBC6s were able to depolarize AII cells enough to cause glycine release and thus cross-over inhibition to these OFF-alpha cells. This cross-over inhibition has been described earlier and thought to operate in photopic conditions Manookin et al., 2008; Munch et al., 2009). Here, we provide the most direct evidence possible to support this case.

Our data clearly suggests that CBC6s→AII cell gap junctions are functional in photopic conditions. While this enables cross-over inhibition to OFF alpha cells, it comes at an expense of dramatic loss of excitability of CBC6s. As we understand, the loss needs to be somehow compensated. In this regard, it was interesting that AII cells were earlier shown to possess voltage-gated sodium channels (Boos et al, 1993). We confirmed it as well by patching AII cells in the retinal slices. Not surprisingly, CBC6s do not appear to have their own voltage-gated sodium channels (Fig 1). However, spikelets produced by AII cells when CBC6s are depolarized appear to propagate to the soma of CBC6s, which were blocked by MFA as expected. It is likely that these ‘’borrowed” spikelets might be larger in magnitude at the bipolar cell axon terminals where they form electrical synapses with AII cell arboreal dendrites.

More importantly, are these ‘’borrowed” spikelets capable of enhancing glutamate release from CBC6s? Are they able to partially or fully compensate for the shunting effect we described earlier? We sought to answer these questions in two ways. First, we recorded from ON-alpha RGCs in flat-mount retinas in the presence and absence of 1μM tetrodotoxin (TTX), which had completely blocked voltage-gated sodium currents in the AII cells (Fig 1). Absence of TTX resulted into significantly larger EPSC amplitude and shorter time to peak across a wide range of CBC6s depolarization. The time to reach half-maximal amplitude (EC50) was also significantly faster (Fig 5). In addition, the stimulus that produced paired-pulse facilitation when TTX was superfused caused paired-pulse depression when TTX was absent (Figs 2, 6). Altogether, these data implies that voltage-gated sodium currents enhance the release probability of glutamate from CBC6 terminals, thus enhancing the bipolar output to the ON-alpha RGCs. Importantly, the effects of TTX were reproducible both in the presence or absence of inhibition (Fig 2) but not when gap-junctions were blocked (Fig 3), strongly indicating AII cell as a source of this ‘’amplification”. Second, and potentially more convincing evidence was achieved by recording ON-alpha RGCs in retinal slices (Fig 2). When a nearby AII cell was impaled with a sharp electrode carrying QX-314, the EPSC amplitudes of the recorded ON-alpha RGCs almost dropped to fifty percent. Based on these overall observations, we cannot think of anything else other than AII spikelets to have caused the amplification of CBC6s output.

Previous studies indicate that voltage-gated sodium channels are localized solely on OFF lobular appendages of AII cell (Kaneko and Watanabe, 2007; Wu et al., 2011), almost 30-40μm away from the bipolar cell terminals. Such an arrangement might preferentially help AII cell to enhance their glycine release. While our findings suggest that voltage-gated sodium channels allow AII cells to amplify their glycine release (Fig 7), such enhancement is clearly not restricted to the OFF inner plexiform layer. Because gap junctions allow rapid transmission of signals (Veruki and Hartveit, 2002a, b), the proposed site of voltage-gated sodium channels may not be problematic given their short distance and AII being a narrow-field cell. Alternatively, one also cannot completely rule out that AII arboreal dendrites themselves possess voltage-gated sodium channels. Elimination of Nav1.1, the only isoform known to be expressed by AII cell (Kaneko and Watanabe, 2007; Wu et al., 2011), did not abolish voltage-gated sodium currents recorded from every AII cells (Wu et al., 2011). Ongoing work in our lab indicates that ICA121431, a potent inhibitor of Nav1.1, does not completely block sodium currents in AII cells either. In addition, puffing TTX on the OFF sublamina suppressed a large fraction of sodium currents but did not completely abolish them (Tamalu and Watanabe, 2007). A claim that AII cells possess these channels only at their axon initial segment in the OFF layer were based on immunohistochemistry and local puffing of TTX, neither of which are perfect tools. Finally, given an uncanny ability of AII cells to break conventions (Farshi et al., 2016; Meyer et al., 2016; Yadav et al., 2019; Grimes et al., 2022), one cannot also rule out that there are simply more than one types of AII cells, one of which possess voltage-gated sodium channels at their ON terminals.

It is impressive how the ‘’borrowed” amplification almost completely compensates for the shunting-mediated loss. The exception is when these bipolar cells are very minimally depolarized. It is possible that these scenario perhaps reflect mesopic conditions, where rod bipolar input may obviate such amplification (Kuo et al., 2016). Consistent with gap-junction causing shunting, increasing coupling in the AII-ON CB network produced diminished responses in the On-alpha RGCs and applying TTX did not seem to make a substantial difference (Fig 8). It is striking that the amplification mechanism we propose might need a special condition to operate. We speculate that it happens when CBC6→AII gap junction is open enough to cause strong depolarization of AII cells and thus spikelets. A second condition is that AII→AII gap junctions be relatively closed to prevent further shunting. Photopic conditions seems to fulfill both of these criterion (Kothmann et al., 2009; Yadav et al., 2019).

In summary, here we report that electrical synapses between ON-CBs and AII cells operate in photopic conditions and that ON-CBs surprisingly require amplification by AII cells to reliably feed their output to the post-synaptic retinal ganglion cells. This is achieved by the ability of AII cells to produce spikelets when depolarized by ON CBs through gap junctions. It is noteworthy that AII cells are thought to play a critical role in scotopic and mesopic vision. We present a strong case for their special role in daylight vision.

## METHODS

### Animals

Mice were handled in accordance with protocols approved by the UC Berkeley Institutional Animal Care and Use Committee (AUP-2016-04-8700-1) and conformed to the NIH Guide for the Care and Use of Laboratory Animals. CCK-ires-Cre knock-in mice (Jackson 012706, abbreviated CCK-Cre) were used to drive Cre recombinase under the cholecystokinin promoter to label CBC6 cells.. Ai32 mice (Jackson 024109, B6.Cg-Gt(ROSA)26Sor^tm32(CAG-COP4*H134R/EYFP)Hze^/J) were used to drive channelrhodopsin-2/eYFP fusion protein in Cre-expressing CBC6 or RBC cells. The CCK-Cre and Ai32 alleles were always used in mice in a hemizygous state. Mice of both sexes were used interchangeably. Animals were used within 3 days of postnatal day 60 (p60).

### Retinal Dissection

Mice were euthanized via isoflurane exposure and internal decapitation. Retinal dissection was performed with enucleated eyes in oxygenated ACSF (in mM: 119 NaCl, 26.2 NaHCO_3_, 11 Dextrose, 2.5 KCl mM, 1 K_2_HPO_4_, 1.3 MgCl_2_* 6H_2_O, 2.5 CaCl_2_, 293 mOsm/Kg), (5% CO_2_/95% O_2_) in room light conditions with a dissection microscope. For retinal flat-mount experiments, retinas were flattened with 4 relieving cuts. For retinal slice experiments, flattened retinas were mounted on 13 mm diameter 0.45 μm filter paper discs (MF-Millipore) and sliced to a thickness of 250 μm with a Stoelting Tissue Slicer. Retinal slices were rotated 90 degrees and mounted within a vacuum grease well.

### Electrophysiology

All RGC recordings were performed in flat-mount retinas. Recordings taken from BCs and AII amacrine cells were performed in retinal slices. For RGC recordings, retinas were isolated and incubated in ACSF containing hyaluronidase (9,800 U/mL) and collagenase (2,500 U/mL) for 10 minutes to facilitate penetrating of the inner limiting membrane allowing access to RGC somas for patch clamp recording (Schmidt and Kofuji, 2011). Retinas were mounted ganglion cell side up in a recording chamber and held in place with a harp (Warner Instruments), superfused with ACSF oxygenated with 5% CO_2_/95% O_2_ at a rate of 5 ml/minute at 34 degrees C and viewed under DIC optics with an upright microscope (Olympus). Reagents for ACSF were purchased from Fisher Scientific. ON α-RGCs did not visually express ChR2-eYFP in the CCK-ires-Cre mouse x Ai32 mouse lines. EPSCs from ON α-RGCs were completely blocked with the AMPAR antagonist DNQX. EPSCs also had a delay from light flash to onset of about 6 ms. Other RGCs, that were not ON α-RGCs, expressed ChR2-eYFP that exhibited near instantaneous current with light flash with a delay of about 0.2 ms which was insensitive to DNQX.

For retinal slice experiments, slices were made as described and perfused in the same way as flat-mount retinas. Electrodes were made with boroscilicate capillary glass tubing with OD = 1.5 mm, ID = 1.17 mm (Warner G150TF-4). For RGC recording, glass was pulled to a resistance of 5-6 MΩs using a Narishige pipette puller. For BCs and amacrine cell recording, electrodes were made with a resistance of 7-8 MΩs with a Sutter Instruments P1000. For voltage clamp experiments, the pipette solution contained (in mM): 123 Cs gluconate, 8 NaCl, 1 CaCl_2_, 10 EGTA, 10 HEPES, 10 glucose, 5 Mg^2+^ ATP, 5 QX 314 (Tocris Bioscience), 0.01 Alexa 594, pH 7.4 with CsOH, 293 mOsm/Kg. For current clamp experiments in BCs to measure optogenetic responses, the pipette solution contained (in mM): 125 K^+^ Gluconate, 10 EGTA, 10 KCl, 10 HEPES, 4 Mg^2+^ ATP, 0.01 Alexa 594, 293 mOsm/Kg. Alexa Fluor 594 Hydrazide (ThermoFisher Scientific) at 10 μM was used to dye fill all cells during recording for confirmation of cell identity. Whole-cell recordings were sampled at 20 kHz and filtered at 2 kHz with a Multiclamp 700B amplifier, digitized with a 1440a or 1550a Digidata A-D converter and analyzed offline with Axograph X or Clampfit. Recordings with series resistance above 10% of the cell membrane resistance or reached above 30 mΩ were discarded.

To block photoreceptor responses to light, the kainate receptor antagonist ACET (1 μM) and the mGluR6 agonist L-AP4 (10 μM) were added to ACSF. Gabazine (10 μM) and strychnine (10 μM) were also added to block ionotropic GABA and glycine receptors. MFA (100 μM) was used to uncouple gap junctions. Pharmacological agents were purchased from Tocris Biosciences. Optogenetic stimulation of CBC6s and RBCs was provided by a 470 nm LED delivering 2.1X10^8^ photons/s, (Lumencore or CoolLED pE-4000) measured at the plane of the retina with a spectrophotometer (Thor Labs). For Fig. 2 and 6, stimulation intensity was varied by changing the duration of the stimulus from 0.1 to 10 ms. Thus, the number of photons delivered during the optogenetic stimulus ranged from 2.1X10^7^ (100%) to 2.1X10^5^ (1%).

## Data Visualization and Statistics

All reported statistics and diagrams were generated with KaleidaGraph. Retinal and experimental diagrams were created with Biorender.com.
